# Effects of Applying Different Resonance Amplitude on the Performance of the Impedance-Based Health Monitoring Technique Subjected to Damage

**DOI:** 10.3390/s18072267

**Published:** 2018-07-13

**Authors:** Wongi S. Na, Dong-Woo Seo, Byeong-Cheol Kim, Ki-Tae Park

**Affiliations:** Sustainable Infrastructure Research Center, Korea Institute of Civil Engineering & Building Technology (KICT), Gyeonggi-Do 10223, Korea; dwseo@kict.re.kr (D.-W.S.); bckim@kict.re.kr (B.-C.K.); ktpark@kict.re.kr (K.-T.P.)

**Keywords:** electromechanical impedance, piezoelectric transducer, damage detection, structural health monitoring, nondestructive testing

## Abstract

Smart materials such as piezoelectric transducers can be used for monitoring the health of building structures. In this study, a structural health monitoring technique known as the electromechanical impedance (EMI) method is investigated. Although the EMI method has the advantage of using a single piezoelectric patch that acts both as the actuator and as the sensor, there are still many issues to be addressed. To further understand the problem, the performance of the EMI method on a structure subjected to progressive damage at different resonance frequency ranges and peak amplitudes was investigated using three different statistical metrics: root-mean-square deviation (RMSD), mean absolute percentage deviation (MAPD) and correlation coefficient deviation (CCD). Metal plates were used throughout the study. The results acquired could be used to further understand the damage identification performance of the EMI method.

## 1. Introduction

As civil infrastructures age, factors in the surrounding environment eventually cause their components to deteriorate over time, weakening the integrity of the structure. Most of the buildings around us are made from concrete, metals and composite materials, and as such, maintenance has always been a vital issue. There are many non-destructive ways of checking the status of a structure, including acoustic emission, radiographic, ultrasonic, liquid penetrant, eddy-current testing, magnetic-particle, and more. As the number of structures requiring management is increasing and in order to minimize the need for scheduled maintenance, an effective structural health monitoring system is the preferred choice compared to conventional non-destructive methods, The electromechanical impedance (EMI) method is a structural health monitoring technique that uses a single piezoelectric (PZT) transducer to act as both sensor and actuator [1]. The EMI method uses high frequency structural excitation, usually higher than 20 kHz, through a bonded PZT transducer to detect changes in structural mechanical impedance. The application of high frequency ranges means this method can detect local damage up to a few meters depending on the properties of the host structure. When applying the EMI method, a suitable frequency range needs to be manually determined through trial and error. To accomplish this, various frequency ranges are swept in order to search for a range with multiple peaks, and this range is selected to perform the EMI method. This is a vital step, as the absence of any peaks can result in a failure to identify damage as virtually no variations in impedance signatures will be observed, such as in composite structures [2]. Since the existence of these peaks is usually difficult to predict, the effect of different amplitudes on the performance of the EMI method should be investigated to achieve better understanding of the method.

To date, many investigations have been performed regarding the EMI method. In [2], the author showed that it is possible to distinguish between crack damage and debonding damage using a metal disc, which is similar to the concept shown later in this study. The main idea of the study was to create a fixed resonance frequency range and use an averaging technique to distinguish between the two different types of damage. In [3,4,5,6], environmental factors such as temperature variation effect, corrosive solution exposure, and the durability of PZT transducers were investigated and showed that the EMI method has several issues that needed to be resolved. The idea of using steel wire to minimize the temperature effect was proposed in [3]. Here, a PZT transducer was attached at one end of the wire while the other end is attached to a pipe with temperatures increasing up to 300 °C. The results showed that the PZT transducer did not exceed 40 °C. Na et al. investigated the performance of the EMI method on adhesive joints of glass-epoxy composite plates subjected to corrosive solution [4]. The results showed that damage to the adhesive can be monitored with the EMI method. Wandowski et al. [5] conducted experiments to detect delamination of CFRP panels whereby the authors applied a technique to compensate the effects of signature variation due to change in temperature. Yang et al. [6] also investigated the EMI technique subjected to temperature variations. In addition, the durability of the PZT transducers was also tested by measuring the impedance signature for up to 15 months. The results showed that a silicone rubber layer over the PZT transducer reduced the impedance signature changes during this time period.

In Baptista and Filho [7], Bhalla et al. [8], Panigrahi et al. [9] and Wandowski et al. [10], the studies focused on minimizing the cost of performing the EMI method, as an HP4294 impedance analyzer can cost up to US$40,000. This was achieved by using devices such as a function generator, oscilloscope, FFT analyzer and an AD5933 evaluation board. The experimental results have shown that such systems can also measure impedance signatures with promising repeatability and reliability. Wandowski et al. [10] compared the performance of the AD5933 evaluation board with the impedance analyzer, HIOKI IM3570 and the results indicated that the evaluation board showed promising results.

Although there are many more studies that have been conducted related to the EMI method, no investigation has been carried out on the performance of the method when subjected to different amplitude sizes. Thus, it would be important to know how different statistical metrics perform when subjected to different peak amplitudes as the non-model-based EMI method relies heavily on the signature acquired after the attachment of the PZT transducer. In this study, amplitude is decreased by attaching additional PZT transducers in series where various statistical metrics are used to analyze the acquired impedance signatures subjected to damage.

## 2. The EMI Method

The one-dimensional equation proposed by Liang et al. (1994) shows how the EMI method works. In Equation (1), the electrical admittance Y(ω) is a combined function of $Z_{s}\left(\omega \right)$ and $Z_{a}\left(\omega \right)$, which are the mechanical impedance of the host structure and the PZT transducer, respectively. Other variables in the equation, which are I, V, ω, a, $\varepsilon_{33}^{T}$, δ, $d_{3x}$, ${\bar{Y}}_{xx}^{E}$ represent the PZT output current, PZT input voltage, input frequency, geometric constant, dielectric constant, loss tangent, piezoelectric constant and Young’s modulus, respectively.

(1) $$Y\left(\omega \right)=i\omega a\left(\varepsilon_{33}^{T}\left(1-i\delta \right)-\frac{Z_{s}\left(\omega \right)}{Z_{s}\left(\omega \right)+Z_{a}\left(\omega \right)}d_{3x}^{2}{\bar{Y}}_{xx}^{E}\right)$$

**Figure 1:**
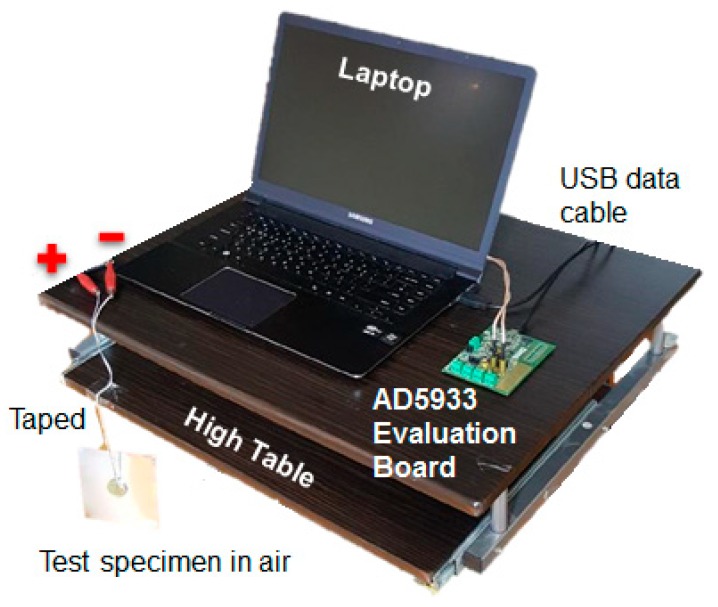
Electromechanical impedance experiment configuration.

To measure the electrical impedance of a PZT transducer, a commercialized AD5933 evaluation board is utilized throughout this study where all the experiments were conducted at a room temperature of 22 ± 0.2 °C. The board is manufactured by Analog Devices Co., Norwood, MA, USA, and retails for less than US$100. The experimental setup is shown in Figure 1. The AD5933 evaluation board is connected to a laptop, which can operate the device using the software provided within the package. The advantage of this device is its weight, as it is very light and its small size makes it portable. It can measure impedance up to 100 kHz with 511 data points and is fully powered by a USB cable that is connected to the laptop. The PZT used for this study was the model PSI-5A4E manufactured by Piezo Systems Inc., Cambridge, MA, USA. The size of the PZT sheet was 72.4 mm × 72.4 mm with a thickness of 0.508 mm, and it was cut into the required sizes for the study. Notice that the wiring of the test specimen is taped onto the edge of the high table to allow it to be left in the air. Since this study involves creating damage to a metal plate, one needs to manually pick up the plate and replace it on the table after creating damage. Leaving the specimen in the air can eliminate any signature variations caused by changes in boundary conditions, as the impedance signatures are extremely sensitive to any changes.

After measuring the electrical impedance of the PZT, the next step is to quantify the intensity of the damage using a statistical method. In this study, three different equations were used, the root-mean-square deviation (RMSD), mean absolute percentage deviation (MAPD) and the correlation coefficient deviation (CCD), which are shown as Equations (2)–(4), respectively. In the equations, ${\left(Z_{k}\right)}_{i}$ represents the reference impedance signature and ${\left(Z_{k}\right)}_{j}$ is the corresponding signature. *N* is the number of impedance signatures with the symbols $\bar{Z}$ signifying mean values and $\sigma_{Z}$ signifying standard deviation. For the study, the real part of the impedance signature was used for data analysis, as it has been experimentally proven to be less sensitive to temperature variations than the imaginary part of the signature [11]. In general, the single value obtained from any of the three equations will be higher with an increase in damage, as the impedance signature will change more severely. The final step in the EMI method is to define a threshold value which is manually defined by experts in this field. Then, any values that exceeds this threshold value is considered to be damaged.

(2) $$\mathrm{RMSD}={\left(\sum_{k=1}^{N}{\left[Re{\left(Z_{k}\right)}_{j}-Re{\left(Z_{k}\right)}_{i}\right]}^{2}/\sum_{k=1}^{N}{\left[Re{\left(Z_{k}\right)}_{i}\right]}^{2}\right)}^{1/2}$$

(3) $$\;\mathrm{MAPD}=\frac{1}{N}\overset{N}{\underset{k=1}{\sum }}\left|\left[Re{\left(Z_{k}\right)}_{j}-Re{\left(Z_{k}\right)}_{i}\right]/Re{\left(Z_{k}\right)}_{i}\right|$$

(4) $$\;\mathrm{CCD}=1-\frac{1}{N\sigma_{Z_{j}}\sigma_{Z_{i}}}\overset{N}{\underset{k=1}{\sum }}\left[Re{\left(Z_{k}\right)}_{j}-Re{\left(\bar{Z}\right)}_{j}\right]\cdot \left[Re{\left(Z_{k}\right)}_{i}-Re{\left(\bar{Z}\right)}_{i}\right]\;$$

## 3. Impedance Signature Peak Reduction Effect on EMI Performance

**Figure 2:**
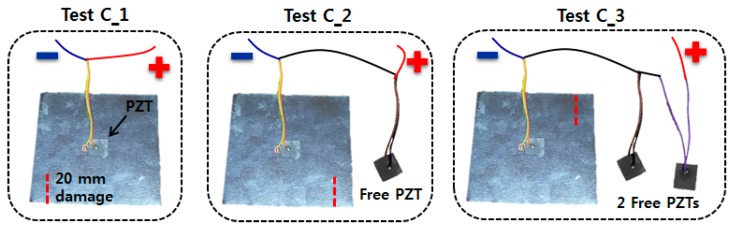
The setup for three test cases for evaluating the EMI performance subjected to amplitude reduction.

To evaluate the performance of the EMI method when the impedance signature is reduced, three 15 mm square PZT transducers were prepared. Three test cases were created and tested, where the first case “C_1” involved attaching one of the three PZT transducers to the center of the 100 mm square metal plate with a thickness 0.3 mm, as shown in Figure 2. This configuration was then connected to the AD5933 evaluation board, and then progressive damage was created using a metal cutter along the dotted line up to 20 mm. During this process, impedance signatures were measured by exciting the PZT transducer at every 2 mm of damage in the frequency range of 25 kHz to 65 kHz in the 80 Hz interval. Here, the frequency range was chosen by sweeping various ranges and selecting the most appropriate range with multiple resonance peaks. Thus, including the reference signature measured before any damage was introduced, 11 impedance signatures were acquired. The second test case “C_2” was conducted by attaching an additional PZT transducer in series to the C_1 configuration (also shown in Figure 2). This additional PZT is left freely in the air, as the attachment will affect the amplitude of the impedance signatures as the equivalent impedance of the two transducers is changed. In this case, since the capacitance of the PZT transducer is directly proportional to its size, the amplitude will decrease. For this second test (C_2), progressive damage is introduced in the same manner. Again, impedance signatures were measured for every 2 mm of damage up to 20 mm in the same frequency range as above, resulting in 11 impedance signatures. The third test case “C_3” involved attaching another PZT transducer in series onto the C_2 configuration, which further increases the overall area of the PZT transducers. Again, progressive damage was introduced along the dotted line and the impedance signatures were measured in an identical manner to the previous two test cases. Through these three experiments (C_1, C_2 and C_3), 33 impedance signatures were acquired in total.

**Figure 3:**
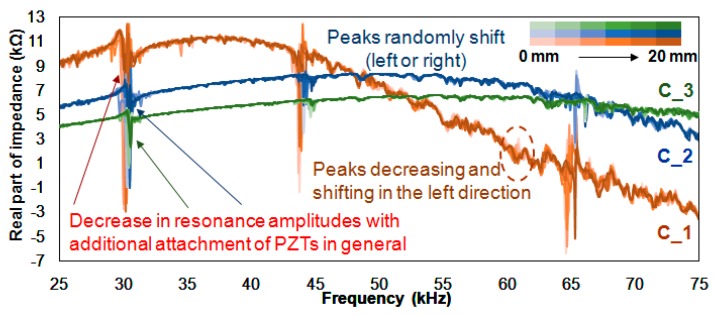
Impedance signatures for C_1, C_2 and C_3 experiment.

Figure 3 shows all the impedance signatures for the three cases where the difference in the height of the amplitudes can be clearly seen. The amplitudes, in general, significantly decrease from C_1 to C_3. The frequency ranges around 30 kHz, 44 kHz and 65 kHz are a good example of this. First, examining the impedance signatures between 28 kHz and 32 kHz, the center of the resonance peaks seems to shift slightly rightward from C_1 to C_3 with the overall amplitudes decreasing, which possibly indicates that progressive damage is changing the resonance frequency of the host structure. For C_1, C_2 and C_3 the maximum peak-to-peak heights of the resonance are around 15 kΩ, 7 kΩ and 5 kΩ, respectively. Next, examining the impedance signatures between 43 kHz and 46 kHz shows a similar result where resonance is shifting rightward as damage progresses. However, the maximum peak-to-peak amplitudes for the three test cases are around 14 kΩ, 5 kΩ and 1.5 kΩ from C_1 to C_3, proving that an increase in PZT area does not have a linear relationship to the maximum height of the peak-to-peak amplitudes. Finally, at the frequency range between 63 kHz and 67 kHz, the resonance is again shifting in the rightward direction with damage. This can be clearly seen in the C_1 test. In addition, another observation made here is the decrease in the peak amplitudes with progressive damage for C_3. The peak that exists at 66 kHz is reduced and virtually disappears after 20 mm of progressive damage.

Using the impedance signatures shown in Figure 3, three statistical metrics (RMSD, MAPD and CCD) were calculated and are displayed in Table 1. The first column represents the damage intensity from 0 mm to 20 mm, the second to fourth columns are the RMSD values (RC_1, RC_2, RC_3), the fifth to seventh columns are the MAPD values (MC_1, MC_2, MC_3) and the last three columns are the CCD values (CC_1, CC_2, CC_3). For the RMSD values, RC_1 starts at 7.38% and ends at 13.53%, RC_2 starts at 5.78% and ends at 7.27%, and RC_3 starts at 1.63% and ends at 4.18%. In addition, averaging the 10 RMSD values for each test cases results in 11.46%, 6.04% and 3.25% for RC_1, RC_2 and RC_3, respectively. These results prove that with smaller amplitudes, the RMSD values will be lower when subjected to the same level of damage. Furthermore, the averaged RMSD values of 6.04% and 3.25% are 53% (6.04/11.46) and 28% (3.25/11.46), respectively.

For the MAPD values, MC_1 is generally larger than the values of RC_1 with an average of 18.42%. The first 2 mm results in a value of 11.51% and increases as the damage progresses, ending with a value of 27.55%. For the MAPD values of MC_2, the average value was significantly decreased to 2.62% (from 18.42%) where the values start from 1.58% and end at 3.46%. This shows that the amplitude reduction of the impedance signature has a vital impact on the MAPD values. Next, the values for MC_3 have an average of 1.64%, starting from 0.71% and ending at 2.12%.

Compared to the RMSD and MAPD values, the CCD values, the results show a different pattern. The highest average value is observed for CC_2 with a value of 5.46%, followed by 3.94% for CC_3 and 1.92% for CC_1. As expected, the CCD index is insensitive to variations in amplitude as it is based on the correlation coefficient. However, when comparing RC_3, MC_3 and CC_3 average values, CC_3 has the highest with 3.94%. This experimentally shows that when there are many peaks with only small amplitudes in the signature, CCD is the preferred choice of the three statistical metrics.

## 4. Creating Various Resonance Frequency Ranges

**Figure 4:**
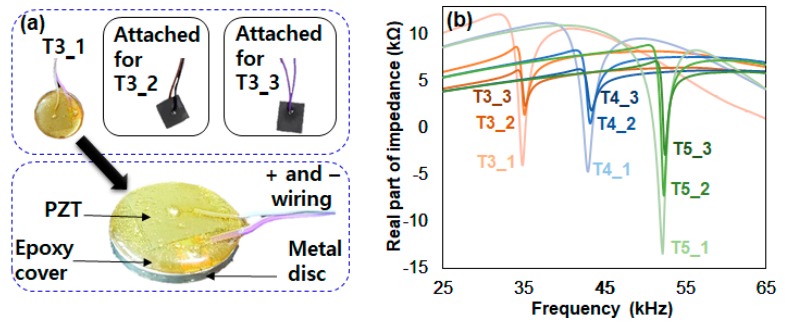
Different PZT attachment (**a**) configuration; (**b**) device impedance signatures.

In the previous section, experiments were used to confirm that a decrease in the amplitude of the impedance signature will also decrease the RMSD and MAPD values (but not CCD values). Thus, to further the investigation, the amplitude reduction effect on the EMI method subjected to damage in various frequency ranges should be examined. To achieve this, the conventional method of attaching a PZT transducer to the surface of the host structure is changed in order to create resonance frequency ranges in various regions. In Na et al. [4], the authors introduced a technique for creating resonance frequency ranges in certain regions regardless of the properties of the host structure. The core of this technique involves attaching a PZT transducer on to a metal disc, then attaching the metal disc onto the host structure. Here, the resonance frequency range can be either increased or decreased simply by changing the thickness of the metal disc. Thus, in this study, this idea is applied to create various frequency ranges where additional free PZT transducers were utilized to reduce peak amplitudes. This configuration can be seen in Figure 4a, where a 15 mm square PZT transducer is attached on top of the metal disc with a 25 mm diameter and 3 mm thickness, then, two more devices were created using two different metal discs with thicknesses of 4 mm and 5 mm. These three devices were used to create 9 test cases referred to as “T3_x”, “T4_x” and “T5_x”, where the first number represents the thickness of the metal in mm and variable x is the total number of PZT transducers connected. For example, T3_1 is the 3 mm metal disc with only the PZT transducer attached, T3_2 is the same with an additional free PZT transducer attached in series, and T3_3 has another free PZT transducer attached to T3_2 in series.

In Figure 4b, the 9 impedance signature measurements are displayed using the three devices that were created. First, examining the three impedance signatures of T3_1, T3_2 and T3_3, there is a decrease in the amplitudes of the resonance peak located between 33 kHz and 38 kHz when additional PZT transducers are attached. Here, it can be said that the resonance is that of the metal disc. The height of the amplitude for T3_1, which was the difference between the highest and lowest points of the impedance signature for this study, is about 16 kΩ. For T3_2 and T3_3, the heights of the peak amplitudes are about 7 kΩ and 3.5 kΩ, respectively. Regarding the other 6 test cases, T4_1, T4_2, T4_3, T5_1, T5_2, and T5_3, the heights of the peak amplitudes are roughly 16 kΩ, 8 kΩ, 4.5 kΩ, 24 kΩ, 16 kΩ, and 10 kΩ, respectively. Overall, the resonance peaks are concentrated in 3 different regions, 33 kHz~38 kHz, 41 kHz~46 kHz and 50 kHz~55 kHz, thus it is possible to investigate the performance of the EMI method subjected to damage at various frequency ranges. Compared to the other cases, the amplitudes are relatively larger for the peaks at the 50 kHz~55 kHz frequency range. Thus, it can be assumed that the metal disc with a thickness of 5 mm might perform better when the EMI method is applied. The results of this test are discussed further later in this paper.

## 5. Conducting the EMI Method with Various Signature Amplitudes

**Figure 5:**
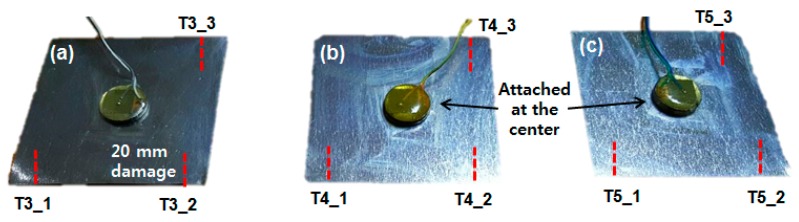
Test setup for (**a**) T3_x; (**b**) T4_x; (**c**) T5_x.

Figure 5 shows the EMI method used to test 9 cases in order to evaluate the damage detection performance using 3 different resonance frequency ranges. Figure 5a shows the three test cases, which will be referred to as T3_1, T3_2 and T3_3 hereafter. Again, the first number represents the thickness of the metal disc, with the second number representing the number of PZT transducers connected for the test. The PZT attached metal disc is adhered to the center of the metal plate using an epoxy glue and was left at room temperature for 24 h to ensure full curing. For the test, damage was created using the same method introduced in Section 2, where a metal cutter was used to cut the metal plate up to 20 mm with impedance signatures being measured at every 2 mm of damage. These three test cases were conducted on the same metal plate as shown in the figure. The damage intensity of each of the cases was assumed to be the same, as the distance from the PZT transducers was identical. For each test case, 11 impedance signatures were acquired in total (including a reference signature) in the frequency ranges between 25 kHz to 65 kHz. Figure 5b, c shows the test specimen for the remaining 6 tests (T4_1, T4_2, T4_3, T5_1, T5_2, and T5_3), where the tests were identical to Figure 5a with different metal disc thicknesses.

**Figure 6:**
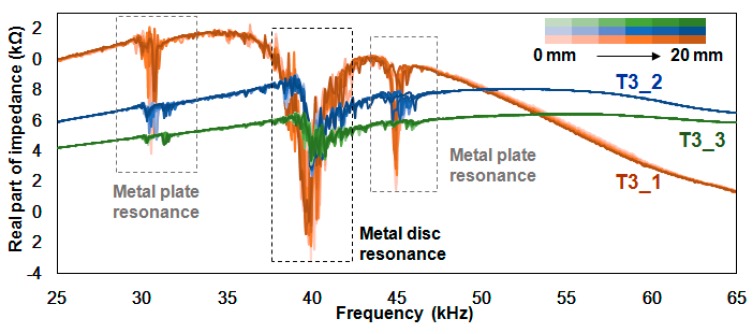
Impedance signatures for the T3_1, T3_2 and T3_3 experiment.

Figure 6 shows the 33 impedance signatures acquired from the T3_1, T3_2 and T3_3 experiments, where resonance peaks are concentrated between 36 kHz and 44 kHz. Compared with the impedance signature before the PZT device was attached onto the metal plate as shown in Figure 4b, one can observe that the resonance frequency has shifted rightward, about 5 kHz. Also, additional resonance frequency ranges have appeared at around 31 kHz and 45 kHz. Since the largest resonance at 36 kHz~44 kHz is the resonance of the metal disc, one can assume that the resonance at 31 kHz and 45 kHz is the resonance of the metal plate. However, regardless of the resonance being either the metal plate or the disc, signature variations can be visually identified in the resonance frequency ranges. Finally, the impedance signature beyond 46 kHz has no resonance where the T3_1 signature shifts in the downward direction subjected to damage. For T3_2 and T3_3, the impedance signatures seemed to be unaffected by damage, showing virtually no sign of any shift movement.

**Figure 7:**
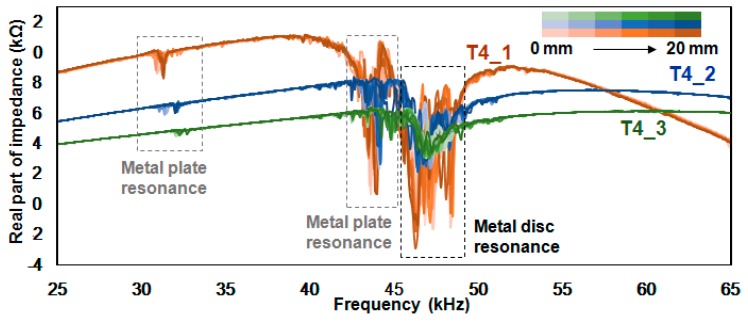
Impedance signatures for the T4_1, T4_2 and T4_3 experiment.

Figure 7 shows the 33 impedance signatures acquired from the T4_1, T4_2 and T4_3 experiments where resonance peaks are concentrated between 43 kHz and 49 kHz. Specifically, , there are two resonance frequency regions which are at 43 kHz~45 kHz and 45 kHz~49 kHz where the first is the resonance of the metal plate as shown in the previous figure. We know that the second resonance is the resonance of the metal disc, as it was experimentally proven that the resonance shifts rightward after the attachment of a metal plate. In addition, resonance is larger with more dynamic activities in the 43 kHz~45 kHz range compared to the previous figure, as the resonance of the metal disc has influenced the outcome. The small resonance located at 30 kHz~33 kHz is smaller when compared to the previous figure, and one possible cause of this is the thicker metal disc used for the experiment, as T4_x is 1 mm thicker than T3_x, thus making the PZT transducer 1 mm further away from the host structure. For the frequency range above 55 kHz where no resonance can be observed, the impedance signatures for T4_1 show a downward shift, with the T4_2 and T4_3 impedance signatures remaining virtually unchanged.

**Figure 8:**
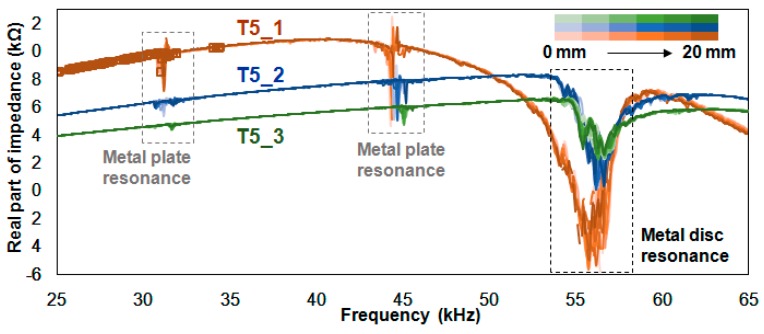
Impedance signatures for the T5_1, T5_2 and T5_3 experiment.

Figure 8 shows the 33 impedance signatures acquired from the T5_1, T5_2 and T5_3 experiments where resonance peaks are concentrated between 53 kHz and 59 kHz. At first glance, the impedance signatures have a relatively smaller number of peaks compared to the previous two figures. As such, one can assume that the values obtained from the statistical methods (RMSD, MAPD, CCD) would be smaller compared to the test cases, T3_x and T4_x. This will be further discussed in Section 6. For the small resonance around 32 kHz, the peaks seem to decrease and almost disappear with the T5_3 signatures. The resonance here is smaller than the resonance observed in the previous figure, as the PZT transducer is further away from the host structure due to the use of a thicker metal disc.

## 6. Results and Discussion

### 6.1. Analyzing the Data for T3_x, T4_x and T5_x Cases

Table 2 shows the values calculated using the three statistical methods for Figure 6. When the values in Table 1 are compared with the C_1, C_2 and C_3 test cases, the averaged values for each column are generally lower. In addition, most of the values increase as the damage progresses. The RMSD values for RT3_1 range from 5.33% to 11.03% with an average value of 9.18%. The average values for RT3_2 and RT3_3 are 2.95% and 2.16%, respectively. This shows that it is best to have impedance signatures with a large amplitude for high RMSD values, as one of the final steps in identifying damage using the EMI method is to define a threshold value. Thus, large RMSD values subjected to damage can allow the user to define a threshold value which can maximize the chance of differentiating a damaged structure from an intact one. The averaged values for MT3_1, MT3_2 and MT3_3 are 13.56%, 1.30% and 0.93%, respectively, which is a similar result to the data in Table 1. Again, a decrease in the amplitudes of the impedance signatures had a significant impact on the MAPD values, as 1.3% is only approximately 1/10 of 13.56%. For the CCD values, the average values are 2.95%, 3.98% and 2.05% for CT3_1, CT3_2 and CT3_3, respectively. This result shows that the reduction in the amplitudes does not have as large an impact, as it did with the RMSD and MAPD values.

Table 3 shows the values calculated using the three statistical methods for Figure 7. With the extra 1 mm thickness of the metal disc, one might expect that the values calculated using statistical methods would be lower compared to the T3_x cases. However, all the averaged values for each column are larger compared to those in Table 2. For the RMSD values, the averaged values for RT4_1, RT4_2 and RT4_3 are 9.52%, 5.61% and 3.28%, respectively. For the MAPD values, the averaged values are 19.11%, 2.33% and 1.31% for MT4_1, MT4_2 and MT4_3, respectively, experimentally proving once again that the height of the peaks of the impedance signatures are very important when using the MAPD method. For the CCD values, the averaged values are 6.15%, 10.00% and 3.58% for CT4_1, CT4_2 and CT4_3, respectively. One of the reasons why the calculated values are relatively larger in this case is the overlap of the resonance of the metal disc and the plate. As shown in Figure 7, this seems to amplify the height of the resonance peaks in the frequency range that corresponds to the resonance of the metal plate.

Table 4 shows the values calculated using the three statistical methods for Figure 8. With the thicker metal disc, one can expect that most of the calculated values will be lower compared to the values in Table 2 and Table 3. However, when we compare the values in Table 4 with those in Table 2, more than half of the averaged values are larger. These are the RMSD averaged values of 3.66%, 3.32%, all the MAPD averaged values of 35.95%, 3.09%, 1.29%, and the CCD value of 2.73%. This shows that while the 5 mm thick metal disc is roughly two times thicker than the 3 mm thick metal disc, this difference does not have too much of an effect on the statistical values. In addition, the averaged value of MT5_1 is 35.91%, approximately triple the averaged value of MT3_1 (13.56%). Next, comparing Table 4 with Table 3, only the three averaged values of RT5_3 (3.32%), MT5_1 (35.95%) and MT5_2 (3.09%) have higher values. For the RMSD and MAPD averaged values, the number decreases from 7.28% to 3.32% and from 35.95% to 1.29%, respectively. For the CCD values, the results are the opposite, as the value increases from 2.06% to 2.73%, regardless of the fact that the impedance signature amplitudes are decreasing.

### 6.2. Regression Analysis on Experimental Data

**Figure 9:**
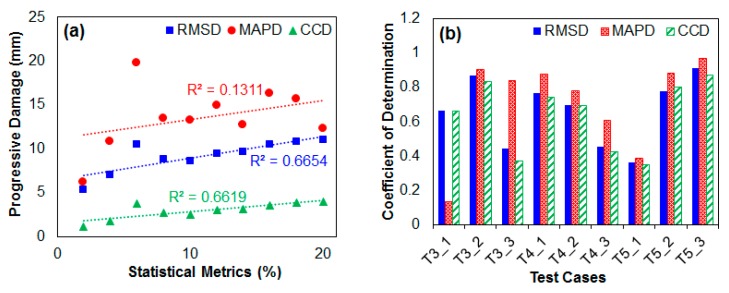
Results for (**a**) T3_1 statistical metric values; (**b**) T3_x, T4_x and T5_x R^2^ values.

In this section, a linear regression analysis is performed using the results from the previous subsection to evaluate the performance of the EMI method subjected to different frequency ranges and statistical metrics (RMSD, MAPD and CCD). Figure 9a shows the scatter plot using T3_1 results from Table 2 (the 2nd, 5th and 8th columns). Three lines of best fit are drawn where the coefficient of determination (R^2^) is 0.67 for RMSD, 0.13 for MAPD and 0.66 for CCD. As damage increases, all three lines show an increasing trend where the slopes have virtually the same steepness. The R^2^ of 0.13 obtained from the MAPD values is considerably lower than the other two statistical metrics, and one of the reasons for this is the randomness of the MAPD values. This randomness can be caused by damage to either a node or an anti-node of the metal plate, which can have a significant effect on the impedance signatures. In Figure 9b, the remainder of the results from the previous subsection are used to plot a bar graph of R^2^ values, where the three bars (RMSD, MAPD and CCD) at T3_1 is of Figure 9a. By observation, while the T3_x, T4_x and T5_x cases have different resonance frequency ranges, it seems that this difference does not have a significant impact on the regression analysis results as it appears to be random. For example, among the T3_x cases, the T3_2 case seems to have highest R^2^ values. Among the T4_x cases, the T4_1 case has the highest R^2^ values, while of the T5_x cases, the T5_3 case has the highest R^2^ values. These results show the complexity of the EMI method data analysis. However, one common feature that can be noted is that the MAPD values are always higher compared to the RMSD and the CCD values, with the exception of the T3_1 case. In addition, the RMSD values are always higher than the CCD values except for the T5_2 case. Overall, the three statistical metrics have similar values for most of the cases, with the T5_3 case having the highest values out of all the values. Another observation that can be made here is that when the impedance signature amplitude decreases from T4_1 to T4_3, the R^2^ values also decrease, whereas the T5_x cases result in the exact opposite (R^2^ values increasing from T5_1 to T5_3). This shows that the size of the amplitudes does not have that significant an effect on the R^2^ values.

## 7. Conclusions

In this study, a structural health monitoring method known as the electromechanical impedance method was investigated, by subjecting a plate to progressive damage under different resonance frequency ranges and peak amplitude heights. The first part of the study consisted of using a square metal plate with a 15 mm square piezoelectric (PZT) transducer attached at the center plate. The test was to create 20 mm of progressive damage with impedance signatures being measured at every 2 mm step, with 11 impedance signatures being acquired in total. This data was then compared with the second test in which the damage created was identical to the first test, but this time with a free PZT transducer attached in series to reduce the amplitudes of the impedance signature peaks. The third test was conducted with damage created identically to the previous tests, with another free PZT transducer attached to the series circuit. From these three tests (C_1, C_2 and C_3), 33 impedance signatures were measured and these were analyzed using three different statistical metrics, which were root-mean-square deviation (RMSD), mean absolute percentage deviation (MAPD) and correlation coefficient deviation (CCD). The results of these tests show that with the decrease in amplitude with the attachment of additional PZT transducers, the RMSD and MAPD values were also decreased in general. However, for the CCD values, this effect was not seen, which experimentally showed that the height of the resonance amplitudes was not a significant factor when analyzing the impedance signature data with a CCD statistical metric. Here, the results show that MAPD is the most appropriate method to use with large amplitudes as MC_1 results in 11.51% increasing up to 27.55% with 20 mm of damage. However, with small amplitudes, CCD performed the best with CC_3 starting from 1.06% increasing up to 5.97% with 20 mm damage.

The second part of the study involved investigating the performance of the EMI method at different frequency ranges. To achieve this, the conventional method of attaching the PZT transducer was changed by using a metal disc, which was first introduced in Na et al. [4]. Three metal discs (T3_1 to T5_3) with different thicknesses were used to create 9 test cases. For each test, progressive damage was introduced in the same manner as in the first part of the study, and three tests were conducted for each metal plate. Afterwards, three statistical metrics were calculated, and showed a similar pattern to the first part of the study. The RMSD and MAPD values generally decreased with a decrease in the resonance amplitudes while the CCD values had no specific pattern, experimentally proving once again that the resonance amplitudes had very little effect on the CCD values. Here, the MAPD method performed the best with largest amplitudes as MT3_1 resulted in 12.34%, MT4_1 with 32.27% and MT5_1 with 45.11% when the test specimen was damaged up to 20 mm. In addition, the CCD method performed the best at 20mm damage with small amplitudes as CT3_3 resulted in 2.76%, CT4_3 with 6.88% and CT5_3 with 5.13%. This demonstrates that it is better to acquire an impedance signature with large amplitudes.

Finally, linear regression analysis on all the RMSD, MAPD and CCD values obtained from the above tests was conducted to evaluate the performance of the EMI method. By fitting a line of best fit on all cases, it was found that the coefficient of determination (R^2^) value was highest for the MAPD values (8 out of 9 cases) compared to both the RMSD and CCD values. In addition, no significant pattern was observed when conducting the EMI method at different frequency ranges, which demonstrates the complexity of analyzing impedance signature data. One of the reasons for this is the sensitivity of the EMI method as many factors can cause the impedance signatures to change. These include the bonding condition of the epoxy adhesive, small differences in the dimensions of the metal plates, small temperature differences, etc. For this reason, research to compensate unwanted signature changes is an important area which should be researched further. However, the knowledge gained from this study can be used to understand the EMI method even further, bringing us one step closer to using this method for practical applications.

## Figures and Tables

**Table 1:** Three statistical metric values for C_1, C_2 and C_3 experiment.

Damage (mm)	RMSD (%)			MAPD (%)			CCD (%)		
	RC_1	RC_2	RC_3	MC_1	MC_2	MC_3	CC_1	CC_2	CC_3
2	7.38	5.78	1.63	11.51	1.58	0.71	0.90	4.66	1.06
4	10.61	3.92	2.29	16.10	1.70	1.03	1.63	2.37	1.90
6	10.06	5.50	2.09	15.07	2.20	1.08	1.49	4.51	1.62
8	10.95	4.72	3.25	15.77	2.33	1.68	1.73	3.37	3.68
10	11.68	5.94	4.19	17.38	2.80	2.05	1.94	5.20	6.05
12	14.24	7.03	3.41	19.58	3.16	1.75	2.77	7.06	4.05
14	13.32	6.43	3.72	18.36	2.96	1.91	2.46	6.07	4.81
16	11.59	6.92	3.84	21.12	2.95	1.97	1.91	6.75	5.10
18	11.22	6.93	3.86	21.79	3.07	2.05	1.81	6.94	5.13
20	13.53	7.27	4.18	27.55	3.46	2.12	2.54	7.64	5.97
Average	11.46	6.04	3.25	18.42	2.62	1.64	1.92	5.46	3.94

Numbers in different colors represent colors in picture.

**Table 2:** Three statistical metric values for the T3_1, T3_2 and T3_3 experiment.

Damage (mm)	RMSD (%)			MAPD (%)			CCD (%)		
	RT3_1	RT3_2	RT3_3	MT3_1	MT3_2	MT3_3	CT3_1	CT3_2	CT3_3
2	5.33	1.82	1.39	6.21	0.69	0.55	1.11	1.56	0.92
4	7.09	2.12	1.18	10.87	0.88	0.55	1.81	2.03	0.73
6	10.53	2.36	2.04	19.77	0.99	0.88	3.73	2.49	1.78
8	8.82	2.86	2.89	13.50	1.28	0.99	2.69	3.51	3.37
10	8.61	3.13	2.21	13.24	1.37	0.86	2.53	4.25	2.04
12	9.41	2.92	2.11	14.91	1.33	0.94	3.00	3.75	1.89
14	9.65	2.93	2.30	12.76	1.33	1.01	3.12	3.74	2.22
16	10.46	3.25	2.58	16.29	1.46	1.15	3.61	4.57	2.72
18	10.87	4.24	2.20	15.66	1.90	1.08	3.88	7.56	2.03
20	11.03	3.83	2.65	12.34	1.79	1.27	4.02	6.31	2.76
Average	9.18	2.95	2.16	13.56	1.30	0.93	2.95	3.98	2.05

Numbers in different colors represent colors in picture.

**Table 3:** Three statistical metric values for the T4_1, T4_2 and T4_3 experiment.

Damage (mm)	RMSD (%)			MAPD (%)			CCD (%)		
	RT4_1	RT4_2	RT4_3	MT4_1	MT4_2	MT4_3	CT4_1	CT4_2	CT4_3
2	5.61	2.86	2.19	8.85	1.13	0.81	2.12	2.46	1.66
4	7.39	2.92	3.57	14.82	1.21	1.27	3.56	2.55	4.14
6	9.38	5.66	3.05	13.76	2.15	1.25	5.69	9.48	2.98
8	8.85	5.67	3.13	15.61	2.34	1.18	5.06	9.14	3.14
10	9.25	6.36	3.27	15.05	2.57	1.37	5.41	11.47	3.43
12	7.67	5.97	3.14	17.54	2.27	1.30	3.81	10.20	3.18
14	9.06	5.49	3.05	18.54	2.23	1.25	5.21	8.68	3.01
16	10.94	5.20	3.30	24.93	2.34	1.37	7.62	7.93	3.52
18	13.28	7.63	3.48	29.70	3.44	1.37	11.12	17.55	3.90
20	13.77	8.30	4.66	32.27	3.60	1.89	11.90	20.56	6.88
Average	9.52	5.61	3.28	19.11	2.33	1.31	6.15	10.00	3.58

Numbers in different colors represent colors in picture.

**Table 4:** Three statistical metric values for the T5_1, T5_2 and T5_3 experiment.

Damage (mm)	RMSD (%)			MAPD (%)			CCD (%)		
	RT5_1	RT5_2	RT5_3	MT5_1	MT5_2	MT5_3	CT5_1	CT5_2	CT5_3
2	5.67	1.66	2.38	22.36	0.89	0.80	1.29	0.61	1.42
4	4.97	3.17	2.24	25.02	1.39	0.85	1.04	1.71	1.29
6	7.09	3.32	2.81	35.83	1.88	1.01	1.92	1.85	1.91
8	8.07	3.49	3.21	43.96	2.15	1.17	2.43	2.02	2.43
10	8.44	3.39	3.25	42.95	2.48	1.26	2.64	1.92	2.50
12	8.39	3.37	3.26	29.65	2.29	1.38	2.62	1.91	2.52
14	6.35	4.35	3.56	26.86	3.43	1.37	1.58	3.06	2.98
16	8.18	4.91	3.52	37.23	5.75	1.45	2.50	3.83	2.91
18	7.44	4.23	4.24	50.54	4.90	1.69	2.09	2.90	4.16
20	8.23	4.68	4.72	45.11	5.73	1.88	2.53	3.50	5.13
Average	7.28	3.66	3.32	35.95	3.09	1.29	2.06	2.33	2.73

Numbers in different colors represent colors in picture.
